# Provider perceptions of systems-level barriers and facilitators to utilizing family-based treatment approaches in adolescent and young adult opioid use disorder treatment

**DOI:** 10.1186/s13722-024-00437-x

**Published:** 2024-03-21

**Authors:** Melissa Pielech, Crosby Modrowski, Jasper Yeh, Melissa A. Clark, Brandon D. L. Marshall, Francesca L. Beaudoin, Sara J. Becker, Robert Miranda

**Affiliations:** 1 Department of Psychiatry and Human Behavior, Warren Alpert Medical School of Brown University, Providence, RI 02912 USA; 2 Center for Alcohol and Addiction Studies, Brown University School of Public Health, Providence, RI USA; 3 Bradley Hasbro Children’s Research Center, Providence, RI USA; 4 Department of Health Services Policy & Practice, Brown University School of Public Health, Providence, RI USA; 5 Department of Epidemiology, Brown University School of Public Health, Providence, RI USA; 6 Department of Emergency Medicine, Warren Alpert Medical School of Brown University, Providence, RI USA; 7 Center for Dissemination and Implementation Science, Feinberg School of Medicine of Northwestern University, Chicago, IL USA; 8 E. P. Bradley Hospital, Riverside, RI USA

**Keywords:** Adolescent, Young adult, Opioid, Opioid use disorder, Treatment, Family, Barriers, Facilitators

## Abstract

**Background:**

Amidst increasing opioid-related fatalities in adolescents and young adults (AYA), there is an urgent need to enhance the quality and availability of developmentally appropriate, evidence-based treatments for opioid use disorder (OUD) and improve youth engagement in treatment. Involving families in treatment planning and therapy augments medication-based OUD treatment for AYA by increasing treatment engagement and retention. Yet, uptake of family-involved treatment for OUD remains low. This study examined systems-level barriers and facilitators to integrating families in AYA OUD treatment in Rhode Island.

**Methods:**

An online survey was administered to clinic leaders and direct care providers who work with AYA in programs that provide medication and psychosocial treatments for OUD. The survey assessed attitudes towards and experiences with family-based treatment, barriers and facilitators to family-based treatment utilization, as well as other available treatment services for AYA and family members. Findings were summarized using descriptive statistics.

**Results:**

A total of 104 respondents from 14 distinct treatment programs completed the survey. Most identified as White (72.5%), female (72.7%), and between 25 and 44 years of age (59.4%). Over half (54.1%) of respondents reported no experience with family based treatment and limited current opportunities to involve families. Barriers perceived as most impactful to adopting family-based treatment were related to limited available resources (i.e. for staff training, program expansion) and lack of prioritization of family-based treatment in staff productivity requirements. Barriers perceived as least impactful were respondent beliefs and attitudes about family-based treatment (e.g., perception of the evidence strength and quality of family-based treatment, interest in implementing family-based treatment) as well as leadership support of family-based treatment approaches. Respondents identified several other gaps in availability of comprehensive treatment services, especially for adolescents (e.g. services that increase social recovery capital).

**Conclusions:**

Family-based treatment opportunities for AYA with OUD in Rhode Island are limited. Affordable and accessible training programs are needed to increase provider familiarity and competency with family-based treatment. Implementation of programming to increase family involvement in treatment (i.e. psychoeducational and skills-based groups for family members) rather than adopting a family-based treatment model may be a more feasible step to better meet the needs of AYA with OUD.

*Trial registration*: not applicable.

**Supplementary Information:**

The online version contains supplementary material available at 10.1186/s13722-024-00437-x.

## Introduction

Evidence-based treatments for opioid use disorder (OUD) exist, yet opioid-related overdoses are impacting adolescents and young adults (AYA) at unprecedented rates, particularly since the onset of the Covid-19 pandemic [1]. From 1999 to 2018, opioid overdose mortality rates in AYA, including those involving both prescription opioids and illicit opioids, increased by 384% [2]. Data also suggests that the opioid overdose crisis is disproportionately impacting youth of color: since 2015, fentanyl-related fatalities increased by 5.1 fold for Black youth and 4.7 fold for multiracial youth versus a 3.5 fold increase among White youth [3]. Although rates of OUD in AYA are relatively low (< 1%), AYA have the highest rates of opioid *misuse* (i.e., use of prescription opioids in a manner other than instructed by a healthcare provider such as taking prescription opioids in higher dosage or longer than recommended by a healthcare provider, using someone else’s prescription, or using opioids to get high) of any age group and the lowest rates of accessing substance use treatment [4]. Specifically, only 6% of adolescents (age 12–17 years) and 7.4% young adults (18–25 years) who need substance use treatment actually receive it [5]. Facilitating AYA engagement and retention in OUD treatment is particularly challenging, especially because medications for OUD (i.e. naltrexone, buprenorphine), which are critical, life-saving treatments, are underutilized with AYA [6]. Among a sample of commercially-insured AYA newly diagnosed with OUD, only 1 out of every 4 youth received medication for OUD [7], suggesting that new approaches to bolster AYA engagement in care are needed.

Family-based treatment (FBT) is one of the most effective and developmentally appropriate psychosocial approaches to treating AYA with substance use disorders [8–10] showing promise for its applications to augment OUD medication treatment [5, 11]. FBT for AYA engages the individual with OUD *and* their primary caregivers/family members in treatment. Of particular relevance to AYA-aged patients, FBT approaches utilize an ecological framework and recognize that many AYA are still developing within and influenced by their family system. This approach aims to influence behavior and interpersonal effectiveness of both parties by leveraging family strengths to bolster support for the individual with OUD (for additional details about the rationale for using FBT approaches with AYA, see Hogue et al. [12]). Although several models and proprietary manuals for FBT exist, these approaches generally crystallize around a set of evidence-based principles best summarized by Hogue et al. [25]: family engagement, relational reframing, family behavior change, and family restructuring. Family engagement in treatment occurs on a continuum [11]. In the absence of an FBT model, families can still be involved in, and supportive of, their loved one’s treatment (herein referred to “family involvement in treatment” [11]). Family involvement in treatment is a more flexible approach for leveraging family support and engagement. For example, family members may participate in collaborative treatment planning or receive education about OUD treatment. See Table 1 for a summary of key aspects of FBT and family involvement in treatment.

**Table 1:** Key terms: defining family-based treatment, family involvement in treatment, and family

	Family-based treatment (FBT)	Family involvement in treatment (FIT)
Definitions *(provided to respondents in the survey instructions)*	**Family-based treatment** involves engaging both the individual with an opioid use disorder and their family members in treatment. Families are active members of treatment, along with the patient. Family strengths are utilized and bolstered to support the individual with OUD	**Family involvement in treatment** is when an identified caregiver or family member is provided with opportunities to be involved in their loved one’s OUD treatment
	**Family:** In this survey, the word “family” refers to biological and non-biological individuals within the patient’s residence and/or who participate in caretaking and guardianship over the patient such as parents, grandparents, siblings	
Level of familyparticipation	High: Family member involvement is required and family members are active and essential participants in treatment. Family engagement is an initial goal of treatment	Variable: Family involvement is typically not required and can be customized based on the patient’s preferences and needs. The level of family involvement may fluctuate over time [depending on the patient’s and/or family’s preference]
Key content/components:	Intervenes at the family-systems level (rather than the individual level). Aims to improve family relations, address family dysfunction that may be contributing to AYA opioid use, and leverage family strengths to support the success of the individual with OUD Evidence-based principles of FBT (summarized by Hogue et al. [25]): • Family engagement • Relational reframing (*aiming to motivate family members to make changes in their relationships)* • Family behavior change *(*via* skills building, coaching, and reinforcement)* • Family restructuring	Can occur in the context of individualized, evidence-based behavioral and psychosocial treatments for OUD **Move this up to the top of the cell
Example activities:	• Family therapy sessions • Skills building to increase emotion regulation, effective family communication, parental monitoring etc • Assess, discuss, and modify family dynamics • Initiate behavioral changes based on family’s goals Models for delivery: Functional Family Therapy [26], Brief Strategic Family Therapy [27], Multidimensional Family Therapy [28]	-Collaborative treatment planning and goal setting -Family education about medication for opioid use disorder -Inviting family members to periodically attend therapy sessions with their loved one -Offering group programming for family members -Sharing updates on patient’s treatment progress with their family

FBT and family involvement in treatment are known facilitators of early engagement in treatment [5, 8, 13] and improved retention [14–16], have strong empirical support [8, 12, 17–20] for addressing substance use among AYA, and yield superior outcomes relative to individual-focused psychosocial interventions. A recent pilot trial of a multi-component intervention targeting medication adherence and treatment engagement in young adults with OUD that included family involvement, showed promise for increasing medication adherence and preventing return to use[5]. Importantly, FBT and family involvement can be facilitated in culturally sensitive ways, and research shows FBT is effective for youth from minoritized racial and ethnic groups [19, 21, 22]. FBT, in particular, also addresses co-morbid mental health and disruptive behaviors in youth, which is important given the high rates of co-morbidity among youth who use substances.

FBT approaches and family involvement are particularly effective for AYA among the ages of 12 to 19 years [8] and they are recommended for use with AYA up to age 25 [12]. In fact, several national organizations recommend FBT as a treatment for AYA substance use (e.g., American Academy of Pediatrics, the National Institute on Drug Abuse [NIDA]) [23]. Despite strong empirical support and promise for their application to OUD treatment specifically, uptake of FBT among clinicians providing adolescent substance use disorder treatment in general, and OUD treatment in particular, remains low [12, 24]. Previous research identified the costs associated with training and implementation, challenges with insurance reimbursement for family-centered services, perceptions about the lack of flexibility of FBT models, and difficulties with long-term sustainability as barriers to successful adoption of FBT for addressing AYA substance use [25]. Of note, proprietary manualized treatments for FBT (i.e., Functional Family Therapy, Brief Strategic Family Therapy) have dominated the market [26, 27]; although these manualized treatments provide an evidence-based road map for delivering FBT, costs for treatment manuals, required trainings, and fidelity/quality assurance requirements are not feasible for many providers and organizations. Importantly, research explicitly measuring provider and systems-level barriers and facilitators to providing FBT is lacking, especially in the context of AYA OUD treatment. Extant work in this area has primarily focused on assessment of patient and family level barriers to couples/partner involvement for adult patients [29–32] with little research focusing on the unique needs of AYA (≤ age 25) populations undergoing treatment [14].

Pre-implementation research to identify barriers and facilitators to the uptake of evidence-based practice is a key component of a comprehensive implementation science research agenda and a necessary first step to improving clinical practice in OUD programs serving AYA. This formative research involves learning the perspectives of individuals who would be involved in or affected by the implementation of an intervention, as well as understanding the relevant processes and culture of the system to inform selection of feasible implementation strategies that address the identified problems [33, 34]. The utility of such formative work is optimized when guided by a theory-driven framework, such as Consolidated Framework for Implementation Research (CFIR; [35, 36]).

The present survey study sought to examine systems-level barriers and facilitators to implementing FBT and opportunities for family involvement in the context of OUD treatment for adolescents and/or young adults by surveying treatment providers and clinic leaders in programs that provide medication and psychosocial treatment for OUD. Due to the developmental differences and distinct treatment regulations for adolescents under the age of 18 and young adults age 18–25, we also examined if perceived barriers and facilitators differed between respondents that work for a program with the capacity to treat adolescents and young adults versus participants that work for a program that only treats young adults. A secondary aim of the survey was to assess treatment resources available for AYA with OUD.

## Methods

### Study design

Participants were recruited for the survey study via direct outreach to local programs that provide medication and psychosocial OUD treatment to adolescents and/or young adults, advertising on local community and professional substance use disorder treatment and advocacy list-servs, as well as snowball sampling (e.g., asking participants to share the study opportunity with colleagues). Survey data were collected online using Qualtrics from January to July 2021. Respondents were first invited to complete a 5-item screener to determine eligibility. To be eligible, participants needed to: (1) work or intern/volunteer as a clinical treatment provider or clinic leader (e.g., administrator) for a program located in Rhode Island that provides both medication (e.g., buprenorphine, methadone) and psychosocial (e.g., counseling) treatment options for OUD. Eligible treatment providers also had to: (1) be involved in providing psychosocial or direct clinical support to patients with OUD; and (2) have young adult **and/or** adolescent patients (age 16–25 years) with OUD on their caseload in the past 12 months. Eligible members of clinic leadership had to: (1) work at an opioid treatment center that provides services to young adult **and/or** adolescent patients (age 16–25 years) with OUD; and (2) be responsible for administrative oversight or supervision of staff. After completing the screener, eligible respondents were automatically directed to the survey. More than one respondent from an organization was eligible to participate in order to capture varying perspectives within an organization. Because this research involved key informants, the research was deemed exempt by the Institutional Review Board. Respondents were compensated for their participation with a $20 Amazon gift card.

### Stakeholder survey on factors influencing family involvement in AYA OUD treatment

The survey was developed by two clinical psychologists (MP and RM) with shared expertise in AYA OUD treatment, FBT, and stakeholder engagement, with consultation from a public health researcher with expertise in questionnaire development (MC). Survey content was informed by knowledge gleaned in key informant qualitative interviews with individuals (n = 30) who have the ability to influence services offered within the opioid treatment system in Rhode Island (e.g. clinic leadership and treatment providers, policy makers, patient advocates; [Pielech et al. in preparation]). Key informants identified barriers and facilitators to utilization of FBT in community opioid programs across the five domains from the original 2009 CFIR model: Intervention Characteristics, Outer Setting, Inner Setting, Characteristics of Individuals Involved, and Implementation Process [35]. Barriers and facilitators to uptake of FBT identified in the interviews became the basis for survey content. Identified barriers were primarily related to the CFIR Inner Setting domain (resources, relative priority, and knowledge access), Individual Characteristics domain (knowledge and beliefs about the intervention, self -efficacy), Outer Setting domain (namely patient needs and resources), and Intervention Characteristics.

The final survey consisted of 63 items across 2 sections. Responses to all questions were optional. Section 1 (37 items) assessed currently available treatment services for AYA with OUD (i.e., case management) or their family members (i.e., working with a family recovery specialist), as well as respondents’ experience with FBT approaches and family involvement in treatment (refer to Table 1 for definitions of family, FBT, and family involvement in treatment that were provided in the survey). Response options to items assessing available treatment services were: “*offered regularly*,” “*offered periodically,”* “*not offered*,” or “*not offered, but needed*.”

Section 2 (26 items) assessed system-level factors that influence family involvement in AYA OUD treatment. Respondents were provided with a list of 26 systems-level barriers and facilitators and asked: “*to what extent do each of the following factors impact your program’s ability to involve families in opioid use disorder treatment for adolescents and young adults, age 16–25 years?*” Response options were: “*does not impact us*,” “*impacts us somewhat*,” and “*impacts us a lot*.” The survey concluded with an open-ended item inviting respondents to share other thoughts or feedback related to identifying and addressing barriers and facilitators to increasing family involvement in OUD treatment for AYA.

At the end of the survey, participants were invited to share basic sociodemographic, professional, and work-related information. Responses to these items were also optional.

The final survey, including sociodemographic items, was reviewed and beta-tested by external content experts to ensure relevance and appropriateness of questions, as well as readability and accessibility. Please refer to the Additional file 1 materials to view a copy of the final survey instrument. 

### Data analysis

Data were exported from Qualtrics to SPSS (version 27) for analysis. Descriptive statistics summarized responses and participant sociodemographic characteristics. Means, standard deviations, percentiles, and ranges were calculated for continuous items; frequencies and proportions were calculated for categorical items. Chi-square tests were performed to examine the potential significance of observed differences in responses between participants that work for a program with the capacity to treat adolescents and young adults versus participants that work for a program that only treats young adults. Open-ended text responses were reviewed to identify relations with close-ended questions as well as any unique, emergent themes.

## Results

### Participants

Of the 153 respondents, 13 (8.5%) did not finish the screening questions and 36 (23.5%) were ineligible, leaving a final sample of 104. Most respondents identified with female gender (72.7%) and were between 25 and 44 years old (59.4%). Regarding racial and ethnic identity, 72.5% of the sample identified as White, 13.2% identified as more than one race, and 9.9% identified as Latinx. Most of the sample had attained a Bachelor’s (38.5%) or Master’s degree (29.7%).

#### Professional characteristics

Respondents (n = 104) reported working for 14 different agencies within the state of Rhode Island that provide opioid treatment for adolescents and/or young adults. The most represented professional disciplines were counseling (37.4%), administration (15.4%), and social work (13.2%). A total of 57.3% of respondents described their current position as direct clinical service providers and 19.1% were program leaders/directors or administrators. Participants reported, on average, being in the profession 10.9 years (*SD* = 12.1) and at their current organization for 4.4 years (*SD* = 4.5 years). Current caseloads ranged from none to 800 (*M* = 87.6 cases; *SD* = 120.7); of note, the highest caseloads were reported by individuals in medical (i.e., nursing) or administrative roles at large treatment programs. Table 2 provides details regarding sociodemographic and work-related characteristics for the sample.

**Table 2:** Respondent sociodemographic and work-related characteristics*

Variable		*N*	%
Age	18–34 years old*	9	31.9%
	35–44 years old	27	29.7%
	45–54 years old	18	19.8%
	55 years and older*	17	18.7%
Sex at birth	Male	22	25.0%
	Female	64	72.7%
Gender identity	Male	22	24.2%
	Female	67	73.6%
	Non-binary / third gender	0	0.0%
Hispanic/ Latine	Yes	9	9.9%
	No	80	87.9%
	Unsure	0	0.0%
	Black or African American, Haitian, or Cape Verdean	5	5.5%
Race	White	66	72.5%
	More than one race	12	13.2%
	American Indian or Alaska Native, Asian, or something else*	4	4.4%
Highest level of education	Some college	10	11.0%
	Associate's degree	7	7.7%
	Bachelor's degree	35	38.5%
	Master's degree	27	29.7%
	Advanced level degree beyond Master’s (e.g. MD, PhD, JD)	9	9.9%
	Other	3	3.3%
Primary professional discipline	Case management	4	4.4%
	Counseling	35	38.5%
	Psychology	5	5.5%
	Social work	12	13.2%
	Nursing	10	11.0%
	Physician	6	6.6%
	Administration	15	16.5%
	Other (includes peer or family recovery specialists)*	6	6.6%%
Current job role	Program director or administrator	17	19.1%
	Clinical supervisor	6	6.7%
	Direct clinical service provider (e.g. nurse, counselor, doctor, social worker)	51	57.3%
	Support staff	12	13.5%
	Other	3	3.4%
Length of time in profession	M = 10.9 years (SD = 12.1 years)		
Length of time at current organization	M = 4.4 years (SD = 4.5 years)		
Current caseload	M = 87.58 cases (SD = 120.68)		
	None or N/A	10	11.4%
	Less than 10 cases	6	5.8%
	10–25 cases	11	12.5%
	26–49 cases	11	12.5%
	50–75 cases	24	27.3%
	76–99 cases	6	6.8%
	100 or more	19	21.6%

^*^To protect respondent anonymity, categories of potentially- identifying demographic characteristics with less than 5 respondents were collapsed

### Treatment services for AYA with OUD

Although all respondents worked for programs that provide treatment to young adults, only 25% of respondents reported that their program has the capacity to provide OUD treatment to adolescents aged 17 years or younger. Approximately 13.5% of the sample reported not knowing whether their program could treat adolescents. A small subset of respondents (11.1%) stated that their program was planning to expand treatment services to adolescents in the future.

Services for AYA with OUD most commonly offered included individual counseling, case management, pharmacotherapy, group counseling, and psychiatric medication management. Table 3 presents additional details about services offered for AYA across the state. Services for AYA with OUD most frequently endorsed by respondents as “*not offered, but needed*” included: sober social events, vocational counseling/training, yoga, nutrition or dietary counseling, mindfulness/meditation, and legal counseling.

**Table 3:** Treatment service availability for adolescents and young adults in your program

Intervention/programming for AYA patients	Offered regularly		Offered periodically		Not offered		Not offered, but needed		
	*n*	%	*n*	%	*n*	%	*n*	%	Total *N* (denominator)
12 step groups	22	21.6%	13	12.7%	42	41.2%	25	24.5%	102
Behavioral &/or non-pharmacological pain management	46	45.2%	20	19.6%	18	17.6%	18	17.6%	102
Case management	78	76.5%	16	15.6%	2	2.0%	6	5.9%	102
Exercise/ physical fitness	11	10.9%	17	16.8%	50	49.5%	23	22.8%	101
Group counseling	58	56.8%	31	30.4%	2	2.0%	11	10.8%	102
Individual counseling	96	93.2%	6	5.8%	0	0.0%	1	1.0%	103
Legal counseling	5	4.9%	18	17.6%	54	52.9%	25	24.6%	102
Mindfulness/ meditation	22	21.6%	41	40.2%	16	15.7%	23	22.5%	102
Nutritional or dietary counseling	12	11.8%	33	32.4%	31	30.4%	26	25.5%	102
Pain management (medical /pharmacological)	33	32.4%	26	25.5%	27	26.5%	16	15.7%	102
Pharmacotherapy for opioid use disorder	81	79.4%	10	9.8%	7	6.9%	4	3.9%	102
Primary care services	25	24.3%	17	16.5%	41	39.8%	20	19.4%	103
Psychiatric medication management	50	49.0%	21	20.6%	23	22.6%	8	7.8%	102
Sober social events	10	9.8%	23	22.5%	36	35.3%	33	32.4%	102
Vocational counseling/ training	13	12.7%	21	20.6%	34	33.4%	34	33.3%	102
Working with a peer recovery specialist	37	35.9%	31	30.2%	19	18.4%	16	15.5%	103
Yoga	8	7.8%	20	19.6%	44	43.2%	30	29.4%	102
Other (free text responses provided): -Crisis management -Urgent walk in care after normal business hours to initiate MOUD -Hep C treatment and testing -STI treatment -Intensive outpatient care -Referrals for acupuncture -Social support/peer support group -Tobacco cessation									

### Opportunities for family involvement in treatment with AYA with OUD

Respondents reported limited opportunities to involve family members in the treatment of AYA patients with OUD in RI (see Table 4). The most commonly available service for family members was access to free educational materials about recovery, endorsed as available by 64.6% of respondents, followed by communication with family about a patient’s progress and a psychoeducation group, both endorsed by 35.7% of respondents. The most commonly endorsed services that respondents described as *“not offered, but needed”* were increased group therapy options for family members (specifically a skills-building group and a support group), as well as access to working with a family recovery specialist (someone with lived experience with a loved one who uses drugs). An equal percentage of respondents (28.6%) endorsed a crisis support line for family members as “*offered regularly”* or “*not offered, but needed*,” reflecting variations in services available across programs.

**Table 4:** Availability of opportunities to involve family members of adolescent and young adult patients in the patient's OUD treatment

Intervention/programming for families	Offered regularly		Offered periodically		Not offered		Not offered, but needed		
	*n*	%	*n*	%	*n*	%	*n*	%	Total *N* (denominator)
Access to free education materials about recovery	64	64.6%	29	29.3%	3	3.0%	3	3.0%	99
Communication with family about patient's progress	35	35.7%	48	49.0%	11	11.2%	4	4.1%	98
Community outings with family members	6	6.1%	11	11.2%	62	63.3%	19	19.4%	98
Crisis support line for family to use for their loved one	28	28.6%	17	17.3%	25	25.5%	28	28.6%	98
Family therapy sessions	22	22.4%	46	46.9%	14	14.3%	16	16.3%	98
In home family therapy sessions	6	6.1%	5	5.1%	68	69.4%	19	19.4%	98
Individual therapy sessions w/ family members	9	9.2%	26	26.5%	42	42.9%	21	21.4%	98
Motivational speakers	6	6.1%	15	15.3%	47	48.0%	30	30.6%	98
Orientation group (for example: an introduction to the clinic and treatment model)	29	29.9%	21	21.6%	23	23.7%	24	24.7%	97
Psychoeducation group (learning about addiction, treatment, and recovery)	35	35.7%	25	25.5%	12	12.2%	26	26.5%	98
Skills group (to learn skills to help facilitate their loved one’s recovery)	20	20.4%	20	20.4%	22	22.4%	36	36.7%	98
Support group (to process and share experiences related to having a loved one who using drugs)	15	15.2%	22	22.2%	24	24.2%	38	38.4%	99
Telehealth/ sessions with family members	24	24.2%	32	32.3%	23	23.2%	20	20.2%	99
Working with a family recovery specialist (someone with lived experience with a loved one who uses drugs)	12	12.2%	21	21.4%	30	30.6%	35	35.7%	98

### Familiarity and interest in FBT

Respondents’ experiences delivering FBT varied widely: 54.1% had never delivered FBT, 42.2% delivered FBT in their current position, and 24.5% delivered FBT in a former position. When asked about current FBT offerings at their program, 18.4% reported it is “*offered regularly”* to AYA patients, 29.6% reported it is “*offered periodically*,” and the remaining half reported it is “*not offered”* (25.5%) or “*not offered, but needed”* (26.5%). Experience with the provision of family-involvement in treatment more broadly was not assessed.

### Systems-level barriers and facilitators to increasing family involvement and FBT

#### Most impactful factors

Respondents rated the extent to which potential factors influence their program’s ability to involve families in OUD treatment for AYA. Table 5 provides a summary of responses, organized by CFIR domains and subdomains. Most notably, factors perceived as having the greatest impact were CFIR Inner Setting factors related to “available resources” (from the subdomain: readiness for implementation) and Intervention Characteristics related to “costs.” Specifically, more frequently identified barriers included lack of staff availability to lead groups for families (endorsed as *impacts us a lot* by 42.7% of respondents and *impacts us somewhat* by 30.2%), lack of staff trained in FBT (endorsed as *impacts us a lot* by 31.3% of respondents and *impacts us somewhat* by 52.1%), lack of knowledge regarding how to involve family members in treatment (endorsed as *impacts us a lot by* 21.9% and *impacts us somewhat* by 56.3%), issues with insurance reimbursement for services for family members (endorsed as *impacts us a lot* by 26.3% and *impacts us somewhat* by 38.9%), and lack of funding to expand services to families (endorsed as *impacts us a lot* by 40.6% of respondents and *impact us somewhat* by 41.7%). Lack of prioritization of FBT in staff productivity requirements (CFIR domain: Implementation Climate, subdomain: organizational incentives and rewards) was endorsed as *impacts us a lot* by 41.7% of respondents and *impacts us somewhat* by *35.4%.* Relatedly, lack of time in staffs’ schedule for family sessions was endorsed as *impacts us a lot* by 34.4% and *impacts us somewhat* by 42.7%. Open-ended responses echoed survey responses identifying challenges with resources and reimbursement. For example, one clinician wrote: *“Medicaid reimbursement rates leave the programs struggling to expand services, as the reimbursement rates are not commensurate with the increase in operating costs for adding new programs (such as youth or family programs).”*

**Table 5:** Extent to which these factors influence the program's ability to involve families in opioid use disorder treatment for AYA

CFIR domains, subdomains, factors	Total sample						Respondents who work for programs that can treat adolescents & young adults						Respondents who work for programs that ONLY treat young adults**					
	None		Somewhat		A lot		None		Somewhat		A lot		None		Somewhat		A lot	
	*n*	%	*n*	%	*n*	%	*n*	%	*n*	%	*n*	%	*n*	%	*n*	%	*n*	%
**Intervention characteristics**																		
** Evidence Strength & Quality**																		
●Staff do not consider family involvement to be effective for AYA	60	63.20%	27	28.40%	8	8.40%	13	54.2%	7	29.2%	4	16.7%	47	66.2%	20	28.2%	4	5.6%
●Staff do not consider family-based treatment to be effective for AYA	61	64.20%	28	29.50%	6	6.30%	13	54.2%	9	37.5%	2	8.3%	48	67.6%	19	26.8%	4	5.6%
** Costs**																		
●Lack of funding for staff to attend training for family-based approaches	23	24.00%	38	39.60%	35	36.50%	4	16.7%	12	50.0%	8	33.3%	19	26.4%	26	36.1%	27	37.5%
●Issues with insurance reimbursement for services for family members	33	34.70%	37	38.90%	25	26.30%	5	20.8%	9	37.5%	10	41.7%	28	39.4%	28	39.4%	15	21.1%
**Inner setting**																		
** Culture**																		
●The culture of adult treatment models does not fit with family-based treatment models	38	40.00%	41	43.20%	16	16.80%	9	37.5%	10	41.7%	5	20.8%	29	40.8%	31	43.7%	11	15.5%
**Implementation climate**																		
*** Organizational incentives & rewards***																		
●Family treatment is not prioritized in staff productivity requirements	22	22.90%	34	35.40%	40	41.70%	6	25.0%	9	37.5%	9	37.5%	16	22.2%	25	34.7%	31	43.1%
*** Relative priority***																		
●Staff lack of interest in increasing family involvement in treatment	48	51.10%	29	30.90%	17	18.10%	10	41.7%	6	25.0%	8	33.3%	38	54.3%	23	32.9%	9	12.9%
●Staff lack of interest in family-based treatment approaches*	49	51.60%	30	31.60%	16	16.80%	11	45.8%	4	16.7%	9	37.5%	38	53.5%	26	36.6%	7	9.9%
**Readiness for implementation**																		
*** Leadership engagement***																		
●Staff not receiving support and encouragement from the agency for increasing family-involvement in treatment	32	33.70%	46	48.40%	17	17.90%	8	33.3%	11	45.8%	5	20.8%	24	33.8%	35	49.3%	12	16.9%
●Program leaderships’ lack of interest in increasing family involvement in treatment	48	50.50%	31	32.60%	16	16.80%	10	41.7%	8	33.3%	6	25.0%	38	53.5%	23	32.4%	10	14.1%
●Program leaderships’ lack of interest in increasing family involvement in treatment	48	50.5%	31	32.60%	16	16.80%	10	41.7%	8	33.3%	6	25.0%	38	53.5%	23	32.4%	10	14.1%
*** Available resources***																		
●Lack of staff availability to lead groups for families	26	27.10%	29	30.20%	41	42.70%	4	16.7%	6	25.0%	14	58.3%	22	30.6%	23	31.9%	27	37.5%
●Lack of funding to support expansion of services to families	17	17.70%	40	41.70%	39	40.60%	3	12.5%	7	29.2%	14	58.3%	14	19.4%	33	45.8%	25	34.7%
●Insufficient space for family groups to meet	32	33.30%	31	32.30%	33	34.40%	8	33.3%	9	37.5%	7	29.2%	24	33.3%	22	30.6%	26	36.1%
●Insufficient space for family therapy sessions	31	32.30%	38	39.60%	27	28.10%	7	29.2%	12	50.0%	5	20.8%	24	33.3%	26	36.1%	22	30.6%
●Lack of time in staffs' schedule for family sessions	22	22.90%	41	42.70%	33	34.40%	4	16.7%	10	41.7%	10	41.7%	18	25.0%	31	43.1%	23	31.9%
●Lack of time for staff to attend trainings for family-based approaches	30	31.30%	34	35.40%	32	33.30%	6	25.0%	8	33.3%	10	41.7%	24	33.3%	26	36.1%	22	30.6%
●Lack of staff trained in family-based treatment	16	16.70%	50	52.10%	30	31.30%	3	12.5%	11	45.8%	10	41.7%	13	18.1%	39	54.2%	20	27.8%
*** Access to knowledge and information***																		
●Lack of staff knowledge regarding how to involve family members in opioid use disorder treatment for AYA patients	21	21.90%	54	56.30%	21	21.90%	4	16.7%	11	45.8%	9	37.5%	17	23.6%	43	59.7%	12	16.7%
●Staff are unsure of how to document family sessions	46	47.90%	34	35.40%	16	16.70%	9	37.5%	8	33.3%	7	29.2%	37	51.4%	26	36.1%	9	12.5%
**Characteristics of individuals**																		
** Knowledge & Beliefs about the Intervention**																		
●Staff concerns that families enable loved ones substance use*	33	34.70%	44	46.30%	18	18.90%	10	41.7%	6	25.0%	8	33.3%	23	32.4%	38	53.5%	10	14.1%
●Lack of staff who are comfortable working with patients and their family together	35	36.50%	43	44.80%	18	18.80%	7	29.2%	11	45.8%	6	25.0%	28	38.9%	32	44.4%	12	16.7%
●Staff concerns that family involvement in treatment will perpetuate enabling dynamics	43	45.30%	37	38.90%	15	15.80%	14	58.3%	5	20.8%	5	20.8%	29	40.8%	32	45.1%	10	14.1%
●Staff beliefs that family-based treatment is not in the best interest of AYA patients	56	58.90%	33	34.70%	6	6.30%	10	41.7%	11	45.8%	3	12.5%	46	64.8%	22	31.0%	3	4.2%
** Individual stage of change**																		
●Staff resistance to changing clinical practice to increase family involvement	41	43.20%	32	33.70%	22	23.20%	8	33.3%	6	25.0%	10	41.7%	33	46.5%	26	36.6%	12	16.9%
** Other personal attributes**																		
●Lack of staff motivation to increase family member involvement in treatment*	45	47.40%	42	44.20%	8	8.40%	9	37.5%	10	41.7%	5	20.8%	36	50.7%	32	45.1%	3	4.2%

^**^Includes respondents who reported that their program does not have the capacity to treat adolescents as well as respondents who were unsure (N = 14 if their program has the capacity to treat both adolescents and young adults, as it was presumed that those respondents could not reliably report on factors influencing service delivery to adolescents

^*^ p < .05 for chi-square tests of the significance of observed between group differences

#### Least impactful factors

For nearly two-thirds of respondents, Intervention Characteristics factors related to “evidence strength & quality” (e.g., perceived effectiveness of FBT or family involvement in treatment) were rated as not impacting their program’s ability to involve families in OUD treatment for AYA. “Relative priority” and “Leadership Engagement” factors (e.g., staff and program leadership’s lack of interest in increasing family involvement in treatment) were rated as *does not impact us* by half of respondents. Staff beliefs that FBT is not in the best interest of AYA patients was rated as *does not impact us* by 58.9% of the sample.

#### Factors with mixed impact

Some factors received mixed ratings, especially factors in the “Characteristics of Individuals Domain.” For example, “lack of staff motivation to increase family member involvement in treatment” was rated as *impacts us a lot* or *somewhat* by 52.8% and *does not impact us* by 47.4% of the sample. Staff concerns that family involvement in treatment will perpetuate enabling dynamics was rated as *very* or *somewhat impactful* by 54.7% of the sample yet *not impactful* by 45.3%. Reponses to items related to sufficiency of space for family groups/family sessions and time for staff to attend trainings for FBT approaches were divided almost evenly across all three levels of impact.

#### Exploratory analysis: differences in perceived impact of factors from respondents who work for programs that treat adolescents and young adults versus respondents who work for programs that only treat young adults

Three factors differed significantly in perceived impact between respondents who work for programs that treat adolescent and young adults versus respondents who work for programs that only treat young adults. Providers who work for programs that treat only young adults were significantly more likely than those who treat adolescents and young adults to endorse concerns about families enabling substance use as impactful (*X*^2^ [2, N = 95] = 7.1, *p* = 0.03). Providers who work for programs that treat both adolescents and young adults identified staff lack of interest in FBT (*X*^2^ [2, N = 95] = 10.6, *p* < 0.01 and lack of staff motivation to increase family member involvement in treatment *X*^2^ [2, N = 95] = 6.58, *p* = 0.04 as more impactful barriers than those who only treat young adults.

#### Open ended feedback

Open-ended response content (provided by n = 28 unique respondents) included expression of interest and enthusiasm for FBT approaches, as well as acknowledgement of challenges with AYA patient and family engagement. One clinic leader shared*, “our clinic tried to start a family support group with some funding we secured... Unfortunately, the group was VERY poorly attended and ultimately it did not make sense to continue. We continue to look for ways to incorporate families into treatment.... We are sure that there is a need!”* Another stated, *“42 cfr part 2* [a federal law that governs confidentiality of substance use-related treatment records] *and fear of breaching confidentiality drives lack of family engagement.”* Restrictions for providing medication for opioid use disorder (i.e., methadone) to patients under age 18 were also noted as barriers to expanding services to adolescents: *“The legalities related to medicated assisted treatment make it difficult to offer such services to individuals 17 and younger.”*

## Discussion

To our knowledge, this study is the first theory-driven examination of systems-level barriers and facilitators to utilizing FBT approaches for AYA in OUD treatment. An additional aim was to gather feedback regarding both the current treatment services available for AYA with OUD and additional services needed for this population. Respondents represented a range of professional disciplines that provide direct care to AYA with OUD, as well as individuals in clinic leadership roles. Findings have important clinical and policy-related implications and can be used to inform FBT and family involvement in treatment implementation efforts to address gaps in availability of evidence-based, developmentally appropriate treatment for OUD in AYA.

### Clinical implications

#### Providers generally perceive FBT and family involvement as helpful for AYA and expressed interested in adopting an FBT approaches, but require more training and support for successful execution

First, findings from the current study revealed that respondents are interested in FBT approaches. For example, barriers perceived as least impactful to FBT utilization were related to the perceptions of the evidence strength and quality of FBT, interest in FBT (i.e. “relative priority”), interest in FBT implementation, and beliefs about FBT being in the best interest of patients. Positivity about FBT and family involvement in treatment was also a common theme in write-in responses. The barriers perceived as most impactful to increasing family involvement in treatment were related to limited available resources, lack of training in FBT, high costs, and balancing productivity with the demands of an FBT approach. 

These results align with findings from previous studies, which indicate that many providers who serve adolescents with substance use disorders find family involvement helpful and important. It is important to note, however, that although enthusiasm for FBT was high in the current sample, most providers had minimal experience delivering FBT to AYA with OUD, and their agencies did not routinely involve families in the treatment process. Given that FBT has a strong evidence base for treating AYA substance use, there is a need for widespread training efforts aimed at providing clinicians who treat AYA with OUD with up-to-date, evidence-based information and skills. 

It is also noteworthy that nearly half of respondents identified concerns that family involvement in treatment would perpetuate enabling dynamics, a barrier that was identified as more impactful amongst respondents that work for programs who only treat young adults. Concerns about FBT leading to “enabling” behaviors may be attributable to knowledge gaps about FBT and are essential to address in provider trainings, especially to young adult treatment providers, to prevent stigma towards families. Labeling family member’s behavior as “enabling” can be perceived as stigmatizing because the term infers blame and judgement of the family member’s actions [37]. This finding is consistent with other work documenting that high levels of stigmatizing views among providers serve as barriers to implementation of effective OUD treatment [38].

#### Increasing opportunities for evidence-based family involvement in OUD treatment, rather than adopting an FBT model, may be more feasible

In line with extant work, barriers to FBT utilization identified in the present study highlight a potential need to rethink how programs can engage families in OUD treatment in a feasible, affordable, sustainable, and effective way [25, 39]. Manualized FBT models, where family members are fully integrated into treatment, are well studied and dominate the market, yet they perpetuate implementation barriers due to the cost of materials, initial licensing, and staff certification (including performance feedback for quality control) [25]. Family involvement in treatment is a broader and more flexible approach that is compatible with evidence-based individualized psychosocial/ behavioral treatments for OUD. Thus, it may be more feasible to focus on increasing opportunities for evidence-based *family involvement* in treatment (i.e. psychoeducation and skills building groups for family members) based on Hogue’s identified core elements of family therapy [25]. Utilizing core principles of FBT when providing opportunities to involve family members allows for some strengths of FBT to be leveraged. Focusing on opportunities for family involvement in treatment would also allow for family members to access resources separately from their child and potentially mitigate concerns regarding patient privacy and confidentiality. For example, one of the primary services for family members that respondents endorsed as “*not offered, but needed*” was increased group therapy options (specifically skills-building group and support groups), which does not require participation of AYA with family members. Previous research demonstrates that skill- and coping-based groups for parents can have positive outcomes in relation to adolescent substance use [40, 41]. Respondents also identified that families may benefit from access to family peer recovery support specialists (i.e., someone with lived experience with a loved one who uses drugs). Although there is little work to date on the extent to which offering families the option to work with a family recovery specialist impacts treatment outcomes for AYA with OUD, this may be a promising avenue given that peer-based recovery services may be helpful for decreasing stigma-related barriers to treatment engagement.

#### Adolescents with OUD and their family members need more developmentally tailored treatment services and providers need more training in delivering developmentally tailored care

Our results also highlight the importance of addressing the unique developmental needs of AYA with OUD, especially adolescents. It is noteworthy that while all respondents in this study were direct service providers or clinic leaders who work with young adults with OUD, most respondents work in programs that primarily serve adult populations. Only 25% of the respondents reported that their program has the capacity to treat adolescents, underscoring the critical service gap for adolescents with OUD.

One potential solution to address gaps in treatment availability is to take advantage of the existing infrastructure of adult OUD treatment programs and expand OUD services to the adolescent age group. Accredited opioid treatment programs that provide only methadone could not expand services to adolescents, as it is not indicated for use with adolescents. However, it would be feasible for programs that provide both methadone and buprenorphine or only buprenorphine to expand their services to adolescents over the age of 16 because buprenorphine is approved for use with youth over age 16. The fact that buprenorphine, one of the leading medications for OUD, is approved for use with youth aged 16 and older is unique amidst the current landscape that lacks any other FDA-approved pharmacotherapy options for adolescents with substance use disorders [42].

Given the unique developmental needs of adolescents with OUD, however, it will be important for programs to adopt new clinical and administrative procedures (e.g., procedures related to billing and confidentiality for patients under age 18, policies around involving caregivers and family members in treatment) to best meet the needs of adolescent clients. It is also essential for providers to receive training in developmentally appropriate, tailored, evidence-based care for this age group and address low motivation to increase family member involvement in treatment, which was rated as a more impactful barrier by respondents who work for a program with the capacity to treat adolescents and young adults. Follow up work is also needed to disentangle factors beyond FDA approval that uniquely impact service delivery to patients under age 18 versus young adults.

### Future research directions

The present results have important implications for future research. Areas of future research should focus on the implementation of FBT and opportunities to involve families in treatment, effectiveness of various opportunities of family-involvement in treatment on youth outcomes, as well as the impact of family recovery specialist services in OUD treatment. This research should utilize hybrid-effectiveness implementation designs to dually evaluate effectiveness of these modified clinical interventions as well the feasibility and success of implementation strategies [43]. Such research would also illuminate whether, relative to FBT, family-involved approaches yield similar effect sizes and/or increased feasibility and sustainability. Given the persistent impact of funding related barriers to the uptake of FBT, it is also recommended that future research investigate the range of funding barriers influencing the use of FBT, as well as the cost-effectiveness of FBT. In particular, we recommend that studies assessing the cost-effectiveness of FBT use standardized methods [44], given that lack of quality economic evaluation in existing FBT research has limited the field’s ability to understand the cost-effectiveness of FBT [45]. Considering economic factors up front could help to increase the feasibility and sustainability of evidence-based practices and enhance understanding of the cost-effectiveness of these approaches.

Although the focus of this study was to understand perceived barriers and facilitators to the uptake of FBT among direct service providers and clinic leaders, an essential next step is to also survey AYA and their families. For example, some participants in the current study noted family members’ lack of engagement as a primary barrier to implementing FBT, and other respondents noted that they often assume AYA do not want their family members involved. It is important to get input directly from those with lived experience, including AYA and their families, to gauge the attitudes of patients and families toward FBT beyond provider's reports and assumptions of families’ willingness to participate [46]. Similarly, it will be important to involve providers and clinical leadership in future studies to better understand clinician’s decision-making regarding involvement of families, determining when and how to use FBT, as well as the association between clinical decisions and patient outcomes.

### Policy implications

If future work finds that modified versions of FBT are effective in OUD programs for AYA, then results of the current study suggest the need for policy changes at multiple levels. First, it is worth noting that when asked about barriers to offering FBT, in addition to identifying their staff's lack of knowledge and training in FBT as barriers, respondents primarily identified barriers related to funding, including issues with insurance reimbursement, and lacking funds to expand services (e.g., pay for additional staff, pay for staff to attend trainings). Nearly two-thirds of participants endorsed challenges with insurance reimbursement for FBT, which is consistent with prior work highlighting the impact of financing models on the implementation of FBT [39]. Neither public nor private insurance typically cover FBT or only reimburse limited amounts that are far from commensurate with the cost of these services. Dopp et al. [39] provide a roadmap for making multi-level (e.g., clinic, payer) policy changes to address funding and reimbursement related barriers and encourage the uptake of FBT. They propose increased collaboration with payers, creation of mechanisms with explicit dedication to paying for family-based services at all levels of care, and integrating FBT with other healthcare because it is often separate from medical or mental health care, creating hurdles for both the patient and clinician [39]. Programs need adequate reimbursement and funding not only for staff time spent delivering FBT, but also for implementation, training, modifying clinic workflows, etc., making reimbursement a significant factor in alleviating cost and resource strain as well as facilitating sustainment of FBT implementation.

Despite several provider respondents noting that the leadership at their respective agencies was interested in increasing family involvement, results indicate that many agencies do not have adequate policies to support the use of FBT. For example, approximately 50% of the respondents indicated that lack of prioritization of FBT in staff productivity requirements was a very impactful barrier and noted difficulties in finding time in their schedules for family sessions. It will be important for agencies who serve AYA with OUD to make policy changes that support the use of FBT, such as policies that consider family sessions as part of a clinician’s billable hours/productivity. Further, to support the training of clinicians, policies could be put in place that offer providers paid time off or a certain amount of professional development funds to pay for training and continuing education. Yet, these trainings must be affordable and flexible in the delivery format, as lack of funding and time for staff training in family-based treatment approaches were frequently endorsed barriers to FBT uptake.

### Strengths and limitations

The current study has several strengths. First, this study utilized a theory-driven approach and focused on provider and systems level barriers, which are often overlooked in implementation science research and planning [47]. Additionally, the current study captured perspectives of participants from multiple disciplines, including psychologists and master’s level clinicians, who worked at diverse community- and hospital-based OUD treatment agencies throughout the state. Further, the project was conducted in a northeastern state at the epicenter of the opioid epidemic where there is an identified need for additional treatment resources for AYA. However, some limitations should be noted. For example, the providers involved in the present study primarily reported working with adults, which may limit their ability to provide suggestions for AYA focused care, especially adolescents. Stratified analyses examining reported barriers amongst respondents that work for a program with the capacity to treat adolescents and young adults versus respondents that work for a program that only treats young adults must be interpreted with caution, as survey questions did not separately ask about factors impacting delivery of care for adolescents versus young adults. Thus, it is impossible to know if respondents were reporting on factors unique to either age group. Additionally, information collected about available treatment services is based on respondent knowledge and awareness of their program’s service offerings. Finally, some respondents were from the same organizations, which may impact independence between observations. To protect respondent’s anonymity, reporting of sociodemographic and professional characteristics was optional and, as a result, not all respondents elected to share their program affiliation and thus could not be accounted for in the analyses. Lastly, findings may not generalize to other geographic locations, although given the paucity of developmentally appropriate treatment resources for AYA [48, 49], these findings are likely informative for national program development efforts.

## Conclusion

As life expectancy in the United States declined for the 3rd year in a row, largely driven by opioid-related fatalities, the urgency of improving access to treatments for this life-threatening condition cannot be overstated. FBT and family involvement in treatment are developmentally appropriate, evidenced-based psychosocial treatments for AYA substance use [8–10] that can be utilized to treat OUD in AYA. But uptake of FBT and opportunities for family involvement in community-based treatment settings remains limited. Guided by the CFIR, we examined systems-level barriers and facilitators to implementation of FBT approaches for AYA with OUD in community treatment programs throughout Rhode Island. Results shed light on modifiable factors that may impede and facilitate the implementation of FBT and family involvement in treatment for AYA with OUD and suggest that FBT may need to be adapted to be feasibly implemented in real-world treatment programs.

### Supplementary Information

**Additional file 1.** Full survey instrument used to assess barriers and facilitators to increasing family involvement in opioid use disorder treatment.

## Data Availability

De-identified datasets used and/or analyzed during the current study are available from the corresponding author on reasonable request.

## Abbreviations

OUD: Opioid use disorder
AYA: Adolescents and young adults
FBT: Family-based treatment
SUD: Substance use disorder
CIFR: Consolidated framework for implementation research
