# Supplementary material for: Provider perceptions of systems-level barriers and facilitators to utilizing family-based treatment approaches in adolescent and young adult opioid use disorder treatment
Source: Addict Sci Clin Pract. 2024 Mar 21;19:20. doi: 10.1186/s13722-024-00437-x (PMC10958911; doi:10.1186/s13722-024-00437-x)
Supplement: Supplementary file 1 — Additional file 1. Full survey instrument used to assess barriers and facilitators to increasing family involvement in opioid use disorder treatment. [file 13722_2024_437_MOESM1_ESM.docx]

Barriers and Facilitators to Increasing Family Involvement in Opioid Use Disorder Treatment

Start of Block: Block 12

**Barriers and Facilitators to Increasing Family Involvement in Opioid Use Disorder Treatment for Adolescents and Young Adults**
 Thanks for your interest in this project!

The purpose of this survey is to learn about factors that impact involving the family members of adolescent and young adult patients (age 16-25 years) in opioid use disorder treatment in Rhode Island.

This survey takes about 15-20 minutes to complete. *If you start the survey and then get interrupted, you can go back to the link and finish it at a later time.*

You may choose to skip any questions. Your answers will be confidential.

 **Compensation:** You will earn a $20 Amazon gift card for your time. You must enter your email address at the end of the survey in order to receive the Amazon gift card.

**Please note:** In this survey, “**adolescents and young adults**” refers to patients between the ages of 16 and 25 years.

In this survey, the word “**family**” refers to biological and non-biological individuals within the patient’s residence and/or who participate in caretaking and guardianship over the patient such as parents, grandparents, romantic partners, siblings, etc.

 **If you have any questions or issues with the survey, such as it freezes or you get kicked out- please email us: InvolveFamilies@Brown.edu or call Dr Pielech at 401 297 3336.**
 
**Thank you so much!**

End of Block: Block 13

Start of Block: Block 4

Does your program have the capacity to provide opioid use disorder treatment to adolescent patients, **age 17 years and younge**r**?**

- Yes (1)
- No (2)
- Don't know (3)

Display This Question:

If Does your program have the capacity to provide opioid use disorder treatment to adolescent patien... = No

To your knowledge, is your program planning to expand services to adolescent patients younger than 18 years old?

- Yes (1)
- No (2)
- Don't know (3)

**The following questions ask about treatment services available for adolescent and young adult patients in the treatment program that you work for.**

Check all treatment services that are available in your program for adolescent and young adult patients between the ages of 16 and 25 years old with an opioid use disorder.   *If a service is not currently offered but you think it is a needed service for adolescent and young adult patients, please select "Not available but needed."*

|  | Offered regularly (1) | Offered periodically (2) | Not offered (3) | Not offered, but needed (4) |
| --- | --- | --- | --- | --- |
| 12-step groups (1) |  |  |  |  |
| Behavioral &/or non-pharmacological pain management (2) |  |  |  |  |
| Case management (3) |  |  |  |  |
| Exercise/ physical fitness (4) |  |  |  |  |
| Group counseling (5) |  |  |  |  |
| Individual counseling (6) |  |  |  |  |
| Legal counseling (7) |  |  |  |  |
| Mindfulness/ meditation (8) |  |  |  |  |
| Nutritional or dietary counseling (9) |  |  |  |  |
| Pain management (medical /pharmacological) (10) |  |  |  |  |
| Pharmacotherapy for opioid use disorder (11) |  |  |  |  |
| Primary care services (12) |  |  |  |  |
| Psychiatric medication management (13) |  |  |  |  |
| Sober social events (14) |  |  |  |  |
| Vocational counseling/ training (15) |  |  |  |  |
| Working with a peer recovery specialist (16) |  |  |  |  |
| Yoga (17) |  |  |  |  |
| Other: Please describe briefly (18) |  |  |  |  |

End of Block: Block 4

Start of Block: Block 7

***Family involvement*** in treatment is when an identified caregiver or family member is provided with opportunities to be involved in their loved one’s opioid use disorder treatment, such as receiving updates on the patient’s treatment progress, invitations to attend therapy sessions and groups with their loved one, and inclusion in treatment planning.

**Check all opportunities available in your program to involve family members of adolescent and young adults patients, age 16-25 years, in the patient’s opioid use disorder treatment.**

**If a service is not currently offered but you think it is a needed service for families of adolescent and young adult patients, please select "Not offered, but needed."**

|  | Offered regularly (1) | Offered periodically (2) | Not offered (3) | Not offered, but needed (4) |
| --- | --- | --- | --- | --- |
| Access to free education materials about recovery (1) |  |  |  |  |
| Communication with family about patient's progress (2) |  |  |  |  |
| Community outings with family members (3) |  |  |  |  |
| Crisis support line for family to use for their loved one (4) |  |  |  |  |
| Family therapy sessions (includes patient + family members) (5) |  |  |  |  |
| In home family therapy sessions (6) |  |  |  |  |
| Individual therapy sessions with family members (no patient) (7) |  |  |  |  |
| Motivational speakers (8) |  |  |  |  |
| Orientation group (for example: an introduction to the clinic and treatment model) (9) |  |  |  |  |
| Psychoeducation group (learning about addiction, treatment, and recovery) (10) |  |  |  |  |
| Skills group (to learn skills to help facilitate their loved one’s recovery) (11) |  |  |  |  |
| Support group (to process and share experiences related to having a loved one who using drugs) (12) |  |  |  |  |
| Telehealth/ sessions with family members (13) |  |  |  |  |
| Working with a family recovery specialist (someone with lived experience with a loved one who uses drugs) (14) |  |  |  |  |
| Other: Please describe briefly (15) |  |  |  |  |

***Family-based treatment*** involves engaging both the individual with an opioid use disorder and his/her family members in treatment. Families are active members of treatment, along with the patient. Family strengths are utilized and bolstered to support the individual with an opioid use disorder.   Please answer the questions below about family-based treatment practices within your organization.

Is **family-based treatment**currently provided to adolescents and young adults, age 16-25 years, in your program?

- Offered regularly (1)
- Offered periodically (2)
- Not offered (3)
- Not offered, but needed (4)

Display This Question:

If If Is family-based treatment currently provided to adolescents and young adults, age 16-25 years, in... Offered regularly Is Selected

Is participation in **family-based** treatment required for adolescent and young adult patients, age 16-25 years?

- Yes (1)
- No (2)

Display This Question:

If Which of the following best describes your position as it relates to treatment for opioid use dis... = Frontline treatment provider/staff

Or Which of the following best describes your position as it relates to treatment for opioid use dis... = Member of clinic leadership

Have you **ever** personally delivered **family-based treatment** to adolescents and young adult, patients age 16-25 years, with opioid use disorders?

- Yes, in my current position (1)
- Yes, but not in my current position (2)
- No (3)

Display This Question:

If Have you ever personally delivered family-based treatment to adolescents and young adult, patient... = Yes, in my current position

Or Have you ever personally delivered family-based treatment to adolescents and young adult, patient... = Yes, but not in my current position

In the past 12 months, have you personally delivered **family-based treatment** to adolescents and young adults (age 16-25 years) with opioid use disorders?

- Yes, in my current position (1)
- Yes, but not in my current position (2)
- No (3)

End of Block: Block 7

Start of Block: Block 11

Below is a list of potential barriers to increasing family involvement and implementing family-based treatment approaches in opioid use disorder treatment for adolescents and young adults that were identified by clinicians, program directors, and policy makers in Rhode Island.
SYSTEMS-LEVEL FACTORS (Part 1)

**To what extent do each of the following factors impact your program’s ability to involve families in opioid use disorder treatment for adolescents and young adults, age 16-25 years?**

|  | Does not impact us (1) | Impacts us somewhat (2) | Impacts us a lot (3) |
| --- | --- | --- | --- |
| Lack of staff trained in family-based treatment (1) |  |  |  |
| Lack of staff knowledge regarding how to involve family members in opioid use disorder treatment for adolescent and young adult patients (2) |  |  |  |
| Lack of staff availability to lead groups for families (3) |  |  |  |
| Lack of time for staff to attend trainings for family-based approaches (4) |  |  |  |
| Lack of funding for staff to attend training for family-based approaches (5) |  |  |  |
| Insufficient space for family therapy sessions (6) |  |  |  |
| Insufficient space for family groups to meet (7) |  |  |  |
| Issues with insurance reimbursement for services for family members (8) |  |  |  |
| Lack of funding to support expansion of services to families (9) |  |  |  |
| Lack of staff who are comfortable working with patients and their family together (10) |  |  |  |
| Lack of time in staffs' schedule for family sessions (11) |  |  |  |
| Family treatment is not prioritized in staff productivity requirements (27) |  |  |  |
| Staff are unsure of how to document family sessions (28) |  |  |  |

SYSTEMS-LEVEL FACTORS (Part 2) **To what extent do each of the following factors impact your program’s ability to involve families in opioid use disorder treatment for adolescents and young adults, age 16-25 years?**

|  | Does not impact us (1) | Impacts us somewhat (2) | Impacts us a lot (3) |
| --- | --- | --- | --- |
| Staff concerns that families enable loved ones substance use (14) |  |  |  |
| Staff concerns that family involvement in treatment will perpetuate enabling dynamics (15) |  |  |  |
| Staff not receiving support and encouragement from the agency for increasing family-involvement in treatment (16) |  |  |  |
| Staff do not consider family involvement to be effective for adolescents and young adults (17) |  |  |  |
| Staff do not consider family-based treatment to be effective for adolescents and young adults (18) |  |  |  |
| Staff resistance to changing clinical practice to increase family involvement (19) |  |  |  |
| Staff beliefs that family-based treatment is not in the best interest of adolescent and young adult patients (20) |  |  |  |
| Staff lack of interest in family-based treatment approaches (21) |  |  |  |
| Staff lack of interest in increasing family involvement in treatment (22) |  |  |  |
| Program leadership lack of interest in family-based treatment approaches (23) |  |  |  |
| Program leaderships’ lack of interest in increasing family involvement in treatment (24) |  |  |  |
| The culture of adult treatment models does not fit with family-based treatment models (25) |  |  |  |
| Lack of staff motivation to increase family member involvement in treatment (26) |  |  |  |

**Almost done! Thanks so much for your effort and time so far.**

**These last few questions ask about your own individual characteristics, as well as how best to contact you to pay you for your participation.**

What is your age?

- 18-24 years old (1)
- 25-34 years old (2)
- 35-44 years old (3)
- 45-54 years old (4)
- 55-64 years old (5)
- 65-74 years old (6)
- 75 years or older (7)

What was your biological sex at birth?

- Male (1)
- Female (2)
- Non-binary / third gender (3)
- Prefer not to say (4)

What is your current gender identity?

- Male (1)
- Female (2)
- Non-binary / third gender (3)
- Prefer not to say (4)

Are you of Hispanic/ Latinx descent?

- Yes (1)
- No (2)
- Unsure (3)
- Prefer not to say (4)

How would you describe your racial background?

- American Indian or Alaska Native (1)
- Asian (2)
- Black or African American, Haitian, or Cape Verdean (3)
- White (4)
- More than one race (5)
- Something else (6)
- Prefer not to say (7)

What is the highest level of education that you have received?

- High school diploma or equivalent (1)
- Some college (2)
- Associate's degree (3)
- Bachelor's degree (4)
- Master's degree (5)
- Advanced level degree beyond Master’s (e.g. MD, PhD, JD) (6)
- Other (7) __________________________________________________

What is the name of the program that you work for?

________________________________________________________________

What is your primary discipline/ profession? Please select the one that best describes you.

- Case management (1)
- Counseling (2)
- Psychology (3)
- Social work (4)
- Nursing (5)
- Administration (6)
- Peer or family peer recovery specialist (7)
- Other (8) __________________________________________________

How long have you been working in your current profession? (in years and months)

________________________________________________________________

Which of the following **best** describes your job type?

- Program director or administrator (1)
- Clinical supervisor (2)
- Direct clinical service provider (e.g. nurse, counselor, doctor, social worker) (3)
- Support staff (4)
- Other (5) __________________________________________________

How long have you worked at your current organization? (in years and months)

________________________________________________________________

Approximately how many clients are in your treatment caseload right now?

________________________________________________________________

Would you like to receive periodic email updates about the findings from this research and planned next steps?

- Yes- please email study updates here: (1) __________________________________________________
- No, thanks (2)

May we contact you in the future about participation in other research studies related to adolescent and young adult substance use?

- Yes (1)
- No, thanks (2)

Please provide the email address where you'd like us to send the Amazon gift card to thank you for your participation.

________________________________________________________________

Please feel free to share any other thoughts or feedback related to identifying and addressing barriers and facilitators to increasing family involvement in opioid use disorder treatment for adolescents and young adults (age 16-25 years).

________________________________________________________________

________________________________________________________________

________________________________________________________________

________________________________________________________________

________________________________________________________________
